# Machine learning application in colon cancer treatment outcome prediction

**DOI:** 10.1038/s41598-026-36917-0

**Published:** 2026-01-24

**Authors:** Hadi Ghasemi, Seyed Vahid Hosseini, Abbas Rezaianzadeh, Ali Reza Safarpour, Hajar Khazraei, Pooneh Mokarram, Mozhdeh Zamani, Seyed Ali Nabavizadeh, Alimohammad Bananzadeh

**Affiliations:** 1 HIV/AIDS Research Center, Institute of Health, Shiraz University of Medical Sciences, Shiraz, Iran; 2 Colorectal Research Center, Shiraz University of Medical Sciences, Shiraz, Iran; 3 Autophagy Research Center, Department of Biochemistry, Shiraz University of Medical Sciences, Shiraz, Iran

**Keywords:** Cancer, Colon cancer, Artificial intelligence, Machine learning algorithm, Artificial neural network, Biochemistry, Cancer, Computational biology and bioinformatics

## Abstract

Colon cancer represents a significant global health burden, accounting for a substantial portion of cancer-related morbidity and mortality worldwide. Many studies have been conducted to predict survival outcomes; however, most of these analyses have been performed predominantly via basic statistical methods. The aim of this study was to perform machine learning techniques to build models for survival prediction in patients with colon cancer. A retrospective review of 764 colon cancer patients treated over a 10-year period facilitated the construction of a detailed dataset containing 44 predictor variables and one dependent variable, the survival status of the patients (alive or dead). The data were randomly split into 80% training and 20% testing sets. Prognostic features from the database were used to build machine learning algorithms, including random forest, logistic regression, XGBoost, gradient boosting, categorical boosting (CatBoost), light gradient boosting machine (LightGBM), multilayer perceptron (MLP), and one-dimensional convolutional neural network (1D-CNN) to predict progressive disease outcomes. Models were validated for sensitivity, accuracy and specificity, with predictive ability assessed by receiver operating characteristic (ROC) curve and area under the curve (AUC) calculations. In terms of model accuracy and precision, almost all algorithms produced similar outcomes; however, among the evaluated models, CatBoost achieved the highest accuracy of 0.813, and the random forest model demonstrated the best precision of 0.727, whereas the logistic regression model exhibited the highest recall of 0.658 on the test set. Our results revealed that the random forest algorithm exhibited the highest AUC of 0.83, demonstrating remarkable efficacy in achieving an optimal balance between sensitivity and specificity. In summary, this research highlights the potential of machine learning models to support personalized and timely interventions for colon cancer patients, ultimately aiming to improve patient care and outcomes.

**Supplementary Information:**

The online version contains supplementary material available at 10.1038/s41598-026-36917-0.

## Introduction

Colon cancer represents a significant global health burden, accounting for a substantial portion of cancer-related morbidity and mortality worldwide^1–3^. Despite advancements in diagnosis and treatment, accurately predicting survival outcomes for patients with colon cancer remains a complex and challenging endeavor. Moreover, the identification and validation of prognostic factors are crucial to improve treatment strategies. In recent years, the advent of Machine Learning (ML) techniques has revolutionized the field of oncology by offering sophisticated tools for prognostic modeling and risk stratification^4,5^. Traditionally, clinicians have used basic statistical analyses, such as descriptive statistics, chi-square tests, t tests, and regression models, which are often implemented through software programs such as SPSS, Microsoft Excel, and STATA, to analyze factors influencing colon cancer survival rates^6,7^. However, these conventional statistical methods are limited in their ability to develop predictive models and generate complex, integrative visualizations^8^. This limitation has led to the widespread adoption of various machine learning approaches, such as random forests, XGBoost, logistic regression, gradient boosting, CatBoost, LightGBM, MLP, and 1D-CNN in this field^9–13^. ML algorithms offer unique capabilities in capturing complex relationships within the data, thereby providing a more comprehensive perspective on survival prediction in patients with colon cancer. In this context, recent reports have demonstrated that integrating gene expression data with clinical parameters can significantly improve prediction accuracy^14^. Notably, the effectiveness of ensemble methods such as random forest, XGBoost, and CatBoost, which achieve accuracies between 80% and 93%, has been shown in various types of cancer survival^15,16^.

Despite these advancements, few studies have focused on localized datasets that reflect country-specific clinical practices and patient characteristics. This study addresses this gap by developing and comparing multiple machine learning and deep learning models using a real dataset, aiming to provide more relevant insights for improving colon cancer outcome prediction in this context. Additionally, studies applying a comprehensive comparison of multiple advanced ML algorithms to Iranian patient data to identify key prognostic factors are lacking. Previous survival prediction studies in colon cancer patients have relied primarily on traditional statistical analyses, which often fail to capture complex nonlinear relationships among multiple variables. This study aims to fill these gaps by implementing and comparing various ML models, including deep learning approaches, to increase prediction accuracy and support personalized treatment strategies. Although machine learning models for colon cancer have been previously developed and analyzed, the influencing factors may vary on the basis of location, lifestyle, and available data. Therefore, it is necessary to build models specific to the Iranian context to determine the factors influencing the outcomes of colon cancer treatment in patients. Additionally, performing variable selection via machine learning methods in the medical domain is beneficial, especially when traditional statistical methods are preferred by clinicians^17,18^. Therefore, the primary objective of this study was to develop, compare, and evaluate the performance of multiple machine learning and deep learning models for predicting survival outcomes in colon cancer patients via retrospective clinical data. Specifically, this research (i) identifies the most influential prognostic factors, (ii) ranks and visualizes feature importance, (iii) compares the predictive performance of traditional ML algorithms and neural networks such as the MLP, and (iv) provides insights to support more personalized and timely interventions. This comprehensive approach contributes to the growing body of literature by addressing the need for localized, explainable, and clinically useful predictive models for colon cancer outcomes in Iran, addressing the limitations of traditional statistical approaches and contributing to more precise, data-driven clinical decision-making.

## Materials and methods

In this study, we implemented a range of machine learning algorithms, including random forest, XGBoost, logistic regression, gradient boosting, CatBoost, LightGBM, MLP, and 1D-CNN. The RF algorithm, for example, operates as an ensemble of decision trees suitable for both supervised and unsupervised tasks. XGBoost, an ensemble of classification and regression trees, is well known for its parallel processing capabilities and high predictive performance. Logistic regression handles various variable types and is widely used for binary classification tasks. Gradient boosting combines multiple weak learners to increase the overall prediction accuracy. CatBoost efficiently handles categorical features and incorporates ordered boosting techniques. The LightGBM uses techniques such as gradient-based one-sided sampling (GOSS) and exclusive feature bundling (EFB) for improved speed and accuracy. The MLP, a type of ANN, is designed to address nonlinear problems via a feed-forward backpropagation structure.

### Study design and data sources

We conducted a retrospective analysis using data from patients diagnosed with colon cancer who underwent surgery at the Colorectal Cancer Research Center at Shiraz University of Medical Sciences. The study protocol was evaluated and approved by the Ethics Committee of Shiraz University of Medical Sciences (IR.SUMS.REC.1402.355), and all methods were carried out in accordance with the guidelines provided by this Ethics Committee. The data were gathered from the Shiraz Colorectal Cancer Surgery registry, a sophisticated electronic database managed by Shahid Faghihi Hospital, which is affiliated with Shiraz University of Medical Sciences in Shiraz, Iran. This registry has meticulously documented patients who have undergone colorectal surgery at this tertiary care center since 2007. The study exclusively included patients with a confirmed diagnosis of colon cancer, as verified by pathological reports from intestinal tissue samples obtained prior to surgery. Following tumor resection, an expert pathologist conducted a thorough examination of each tumor, with the final diagnosis and report being meticulously recorded in the database. Upon admission, patients provided informed consent and their contact information to facilitate comprehensive database entry. The registry team encouraged patients to adhere to regular follow-up visits, thereby ensuring the accuracy and completeness of the database. All the methods were performed and visualized via Python (version 3.11.1) with default parameters.

### Data preprocessing and feature selection

Initially, the dataset contained 83 unorganized variables. After several clinicians at the colorectal cancer research center at Shiraz University of Medical Sciences were consulted, 39 variables deemed less significant for predicting colon cancer survival were removed. The data preprocessing involved importing the remaining 44 variables in a comma-separated format and using the na.omit function to remove rows with substantial missing values. The data preprocessing involved importing the remaining 44 variables in a comma-separated format and using the na.omit function to remove rows with substantial missing values. The resulting clean dataset included 764 patient records, with 43 independent (predictor) variables and 1 dependent (categorical) variable indicating patient survival status (alive/dead). The dataset was carefully cleaned and plausibility checked. Implausible zero entries for numeric laboratory and clinical variables were corrected on the basis of the original medical records. The final minimum values for features such as age, BMI, RBC, hemoglobin (HB), hematocrit (Hct), white blood cell count (WBC), platelet count, tumor size, and involved lymph nodes now accurately reflect the lowest biologically plausible values in the patient cohort. Patients who were lost to follow-up were excluded to ensure accurate survival labeling. Tables 1 and 2 provide detailed summaries of all the variables, including descriptions, value ranges, and proportions for the nominal and numerical features. Therefore, the final dataset was slightly imbalanced, and to mitigate the effects of this imbalance, we applied stratified training/test splitting to preserve class distributions during training and testing, and the class weights were adjusted to compensate for the imbalance and reduce bias toward the majority class.

**Table 1:** Numerical feature characteristics.

Variable Name	Description	Minimum	Mean	Maximum
Age	Age of the patients when they are diagnosed with colon cancer	19	57	97
BMI	Body mass indexes op patients	13.1	22.7	51
RBC	The count of red blood cells of patients’ blood	2.71	3.77	7.78
HB	Concentration of hemoglobin in patients’ blood	4.8	9.85	41.4
Hct	The hematocrit of patient’s blood	4.1	35.79	60.5
WBC	The count of white blood cells of patients’ blood	2.45	8.49	38.8
Platelet	The platelet counts in patients’ blood	30	307.3	979
Size of tumor (cm)	The size of tumor (cm)	0.1	5.152245	27
Involved Lymph nodes	The number of lymph nodes identified as cancerous	1	4.48	33

**Table 2:** Categorical feature characteristics.

Variable name	Description	Value	Proportion (%)
**Gender**	The gender status of the patients	Male	53.8
		Female	46.1
**Familial marriage**	Being child of familial marriage	Yes	25.2
		No	74.7
**Family history of colon cancer**	Presence of colon cancer in family history	Yes	19.3
		No	80.6
**Family history of rectal cancer**	Presence of rectal cancer in family history	Yes	2.1
		No	97.8
**Family history of other cancers**	Presence of other cancers in family history	Yes	32.7
		No	67.2
**Addiction**			
Cigarette	Cigarette smoking by patients	Yes	47.5
		No	52.4
Opium	Using opium by patients	Yes	26.2
		No	73.7
Waterpipe	Using waterpipe by patients	Yes	35.9
		No	64
Alcohol	Alcohol consumption by patients	Yes	4
		No	95.9
**Clinical symptoms**			
Abdominal pain	The status of the patients weathers they have Abdominal pain symptom or not.	Yes	58.6
		No	41.3
Cramp	The status of the patients weathers they have cramp symptom or not.	Yes	15.3
		No	84.6
Constipation	The status of the patients weathers they have constipation or not.	Yes	41.2
		No	58.7
Weight loss	The status of the patients weathers they have lost their weight or not.	Yes	40.9
		No	59
Rectal bleeding	The status of the patients weathers they have constipation or not.	Yes	40.6
		No	59.3
Obstruction	The status of the patients weathers they have bowel obstructions or not.	Yes	15.9
		No	84
**Radiotherapy**	The status of the patients weathers they have been treated with radiotherapy or not.	Yes	5.5
		No	94.4
**Chemotherapy**	The status of the patients weathers they have been treated with chemotherapy or not.	Yes	7.6
		No	92.3
**Differentiation (grading)**	Description of a tumor based on how abnormal the tumor cells and the tumor tissue look under a microscope. It is an indicator of how quickly a tumor is likely to grow and spread.	Well differentiation	62.1
		Poorly differentiation	6.3
		Mucinous type	4.9
		Moderately differentiation	26.5
**Depth of invasion**	Depth of invasion of tumor into the colon tissue layers	Behind Serosa	9.5
		Muscularis	20.2
		Omentum	1.4
		Serosal	65.6
		Sub mucosal	3
**Vascular invasion**	Description of a situation which tumor have a tendency to go into the vascular system	Yes	23.8
		No	76.1
**Neural invasion**	Description of a situation which tumor have a tendency to go into the neural system	Yes	14.6
		No	85.3
**Lymphatic invasion**	Description of a situation which tumor have invasion to the lymphatic system	Yes	26.4
		No	73.5
**Tumor deposit**	Description of the patients with clusters of cancer cells in the soft tissue that are discontinuous from the primary tumor	Free	16.1
		Involved	83.8
**T stage**	Description of the patient’s tumor size and extent of the main tumor; Cancer stage classification	T0	0.6
		T1	2.8
		T2	19.2
		T3	64.6
		T4a	10.5
		T4b	2
**N stage**	Refers to the number of nearby lymph nodes that have cancer;	N0	57.7
		N1a	15.2
		N1b	6.3
		N1c	7.2
		N2a	7.1
		N2b	6.3
**M stage**	Description of the patient’s tumor whether has been metastasized	M1a	8.4
		M1b	0.1
		MX	91.4
**Staging**	Description of the patient’s tumor size and how far it has grown	0	0.8
		I	17.2
		IIA	32.7
		IIB	2.6
		II C	1
		III A	3.4
		III B	25.4
		III C	7.6
		IV A	9.2
**Wall thickening of colon**	Description of the patients with thickened colon wall	Yes	79.3
		No	20.6
**Liver metastasis**	Description of the patients with metastasis to the liver	Yes	17.4
		No	82.5
**Para-aortic lymphadenopathy**	Description of the patients with Para-aortic lymphadenopathy	Yes	10.1
		No	89.8
**Pelvic lymphadenopathy**	Description of the patients with pelvic lymphadenopathy	Yes	32.5
		No	67.4
**Kind of surgery**	The kind of surgery done to the cancer patients.	Conversion	6.8
		Laparoscopy	66.5
		Laparotomy	26.6
**Type of surgery**	The type of surgery done to the cancer patients. The type of surgery depends on the cancer stage and tumor size.	Anterior resection	18.6
		Extended left hemicolectomy	5.9
		High anterior resection	0.7
		Lt. Hemicolectomy	19.6
		Rt. Hemicolectomy	41.7
		Total colectomy	10.6
		Total proctocolectomy	2.8
		Ultra low anterior resection	0.1

### Preprocessing

Prior to model development, a series of preprocessing steps were undertaken to optimize the dataset for analysis. Responses indicating refusal or lack of knowledge were considered missing values. A threshold was established to filter out variables, and participants with substantial missing information variables lacking more than 20% of their data and participants missing crucial data points were removed from the study. Outlier detection was performed via the DBSCAN (density-based spatial clustering of applications with noise) algorithm, a robust technique that identifies and isolates outlier instances on the basis of density estimations without making assumptions about the underlying data distribution. Categorical and ordinal features were one-hot encoded and transformed into a machine-readable numerical format, whereas scalar normalization techniques were applied to numerical variables to mitigate the influence of outliers and ensure equitable feature contributions. Missing data were imputed using the mode for categorical variables and the mean for numerical variables, preserving the integrity of the dataset while enabling the inclusion of all relevant cases. Relevant features were selected on the basis of their potential association with postoperative outcomes in patients with colon cancer, as identified through a comprehensive literature review and consultation with clinical experts. To identify and interpret the most influential features for survival prediction, we applied multiple feature importance techniques. Recursive feature elimination (RFE) was utilized in conjunction with a random forest estimator to eliminate the least important features iteratively and retain the most relevant predictors. Additionally, we extracted built-in feature importance scores from the final trained tree-based models (Random Forest, XGBoost, and CatBoost) on the basis of their split and gain criteria. To further enhance interpretability at the individual prediction level, we computed SHAP (SHapley Additive exPlanations) values for the CatBoost model. SHAP values provide a unified measure of how each feature contributes to increasing or decreasing the model output for each patient. The resulting SHAP summary plot highlights the top predictors and their direction of impact on survival classification.

Our dataset, comprising 764 patient records, presented a significant class imbalance, with 173 patients (22.6%) in the ‘dead’ category and 591 patients (77.4%) in the ‘alive’ category. To prevent our machine learning models from being biased toward the majority class and to improve their ability to accurately predict the less frequent ‘dead’ outcome, we applied the synthetic minority oversampling technique (SMOTE) to the training dataset. This process involves generating synthetic samples for the minority class, ensuring a more balanced representation during model training while rigorously avoiding data leakage by applying SMOTE solely to the training set. To ensure the quality and suitability of our dataset for machine learning, rigorous preprocessing was conducted. Following the removal of variables with more than 20% missing values and rows with substantial missing data, the remaining missing entries were imputed: numerical features utilized mean imputation, whereas categorical features were imputed with their respective modes. Outlier detection was systematically performed via the DBSCAN algorithm, which effectively identified and isolated anomalous data points on the basis of density. Finally, to standardize feature scales and ensure equitable contributions during model training, numerical variables underwent scalar normalization, whereas categorical features were transformed via one-hot encoding.

### Machine learning model development

The selected features were formatted appropriately for machine learning analysis. The following machine learning algorithms were explored for developing predictive models: random forest, XGBoost, gradient boosting, logistic regression, CatBoost, MLP, and LightGBM. The dataset was typically split into training/testing (80/20%) subsets, with the training data used to build the predictive models and the testing data used for model evaluation and performance assessment. To ensure robust model training, minimize bias, and select optimal hyperparameters, 5-fold cross-validation was consistently applied to the training dataset. Appropriate hyperparameter tuning was performed for each algorithm to optimize its performance on the given task. To identify and interpret the most influential features for survival prediction, we applied multiple feature importance techniques. First, we used recursive feature elimination (RFE) with a random forest estimator to rank all the input variables according to their contribution to model performance. Additionally, we extracted built-in feature importance scores from the final trained tree-based models (Random Forest, XGBoost, and CatBoost) on the basis of their split and gain criteria. To further enhance interpretability at the individual prediction level, we computed SHAP (SHapley Additive exPlanations) values for the CatBoost model. SHAP values provide a unified measure of how each feature contributes to increasing or decreasing the model output for each patient. The resulting SHAP summary plot highlights the top predictors and their direction of impact on survival classification.

### Machine learning framework

In this study, we employed several machine learning algorithms to ensure robust comparisons and to identify the most effective predictive models for colon cancer treatment outcomes. Specifically, random forest, XGBoost, gradient boosting, CatBoost, and LightGBM were chosen for their strong performance with structured tabular data and their ability to handle nonlinear relationships and feature interactions. Moreover, logistic regression was selected as a classical baseline because of its interpretability and established use in clinical outcome prediction. We also included an MLP to represent standard artificial neural network architectures for learning complex, nonlinear feature representations. By including several types of algorithms, ranging from interpretable linear models to sophisticated tree ensembles and deep learning architectures, we aimed to comprehensively examine which methodological paradigm offers the best balance of performance and clinical interpretability. All the selected machine learning models were tuned to optimize performance and prevent overfitting. To optimize the performance of each machine learning model, we conducted systematic hyperparameter tuning via a combination of grid search and randomized search, depending on the model. For each algorithm, a range of key hyperparameters was defined on the basis of established guidelines and preliminary tests. Tuning was performed via 5-fold cross-validation, and the combination of hyperparameters yielding the highest average Area under the receiver operating characteristic curve (AUROC) on the training folds was selected as optimal. The final hyperparameter settings for each model are summarized in Supplementary Table 1.We also implemented a baseline lightweight 1D-CNN model. The architecture consisted of two convolutional layers (32 and 64 filters, kernel size = 3), each followed by batch normalization and ReLU activation, and a max-pooling layer. A global average pooling layer was used before a fully connected dense layer (128 neurons, ReLU activation) and a final output layer with sigmoid activation. To mitigate overfitting, dropout (rate = 0.4) was applied, and early stopping was used with a patience of 10 epochs based on validation loss. The model was trained with the Adam optimizer (learning rate = 0.001), batch size = 32, and a maximum of 100 epochs.

To ensure the robustness and generalizability of our machine learning models, we implemented several strategies to mitigate overfitting, a common challenge, especially with complex algorithms such as ensemble methods. Our primary defense involved a strict 80/20 training/testing split of the dataset, ensuring model evaluation on entirely unseen data. Furthermore, hyperparameter tuning for each algorithm incorporates regularization techniques inherent to their design and is often utilized early, stopping with a validation set for gradient-based models. The intrinsic properties of ensemble methods, such as random subsampling in random forest and ordered boosting in CatBoost, also inherently contribute to reduced variance and enhanced generalization capabilities.

### Evaluation metrics

The performance of all the developed machine learning models was rigorously evaluated via a comprehensive suite of metrics derived from the confusion matrix. For a binary classification task (predicting ‘alive’ or ‘dead’), the confusion matrix components are defined as follows:

- True positives (TPs): the number of ‘dead’ patients correctly predicted as ‘dead’.
- True negatives (TN): Number of ‘alive’ patients correctly predicted as ‘alive’.
- False positive (FP): the number of ‘alive’ patients incorrectly predicted as ‘dead’. (Type I error)
- False negatives (FNs): the number of ‘dead’ patients incorrectly predicted as ‘alive’. (Type II error)

The following metrics were calculated to compare the performed ML models:

1. Accuracy: The proportion of total correct predictions (both positive and negative).

Accuracy = (TP + TN)/(FP + FN + TP + TN​).

2. Precision (positive predictive value): The proportion of correctly predicted ‘dead’ instances out of all instances predicted as ‘dead’. It measures the exactness of the model.

Precision = TP/(FP + TP​).

3. Recall (sensitivity or true positive rate): The proportion of correctly predicted ‘dead’ instances out of all actual ‘dead’ instances. It measures the model’s ability to find all positive samples.

Recall = TP/(FN + TP​).

4. Specificity (true negative rate): The proportion of correctly predicted ‘alive’ instances out of all actual ‘alive’ instances. It measures the model’s ability to correctly identify negative samples.

Specificity = TN/(FP + TN​).

5. F1 score: the harmonic mean of precision and recall, providing a single metric that balances both. It is particularly useful when dealing with imbalanced datasets.

F1 score = (2 × Precision × Recall)/(Precision × Recall​).

6. AUROC or AUC: A comprehensive metric that evaluates the classifier’s performance across all possible classification thresholds. It represents the probability that the model ranks a randomly chosen positive instance higher than a randomly chosen negative instance. A higher AUC value indicates better overall discriminative power.

## Results

### Machine learning model performance

The RFE was employed to select the optimal set of features for model development. Table 3 presents the performance metrics of the trained machine learning models, including accuracy, precision, recall, specificity, and F1 score. The results are reported as the mean values with standard deviations obtained through 5-fold cross-validation. Among the evaluated models, CatBoost achieved the highest accuracy of 0.813 (± 0.01) and an F1 score of 0.533 (± 0.05) on the test set. The random forest model demonstrated the best precision of 0.727 (± 0.08), whereas the logistic regression model exhibited the highest recall of 0.658 (± 0.12) on the test set.

**Table 3:** Performance metrics of machine learning models.

Model	Accuracy	Precision	Recall	specificity	F1Score
Random forest	0.800 (± 0.01)	0.727 (± 0.08)	0.233 (± 0.07)	0.972 (± 0.01)	0.344 (± 0.07)
XGBoost	0.798 (± 0.02)	0.577 (± 0.06)	0.456 (± 0.10)	0.901 (± 0.02)	0.506 (± 0.08)
Gradient boosting	0.798 (± 0.01)	0.630 (± 0.06)	0.333 (± 0.04)	0.939 (± 0.02)	0.432 (± 0.04)
Logistic regression	0.731 (± 0.03)	0.443 (± 0.04)	0.658 (± 0.12)	0.753 (± 0.03)	0.527 (± 0.07)
CatBoost	0.813 (± 0.01)	0.643 (± 0.06)	0.520 (± 0.09)	0.917 (± 0.03)	0.533 (± 0.05)
MLP	0.775 (± 0.01)	0.413 (± 0.34)	0.058 (± 0.05)	0.991 (± 0.01)	0.102 (± 0.08)
LightGBM	0.771 (± 0.01)	0.508 (± 0.02)	0.442 (± 0.04)	0.871 (± 0.01)	0.472 (± 0.03)
1D-CNN	0.742 (± 0.02)	0.487 (± 0.05)	0.421 (± 0.07)	0.832 (± 0.03)	0.452 (± 0.06)

As shown in Table 3, certain models, such as random forest and MLP, exhibit relatively high precision but lower recall. This imbalance highlights the models’ tendency to favor correct predictions of the majority (alive) class while underdetecting true positive cases of the minority (dead) class.

### Receiver operating characteristic analysis and model performance

**Fig. 1:**
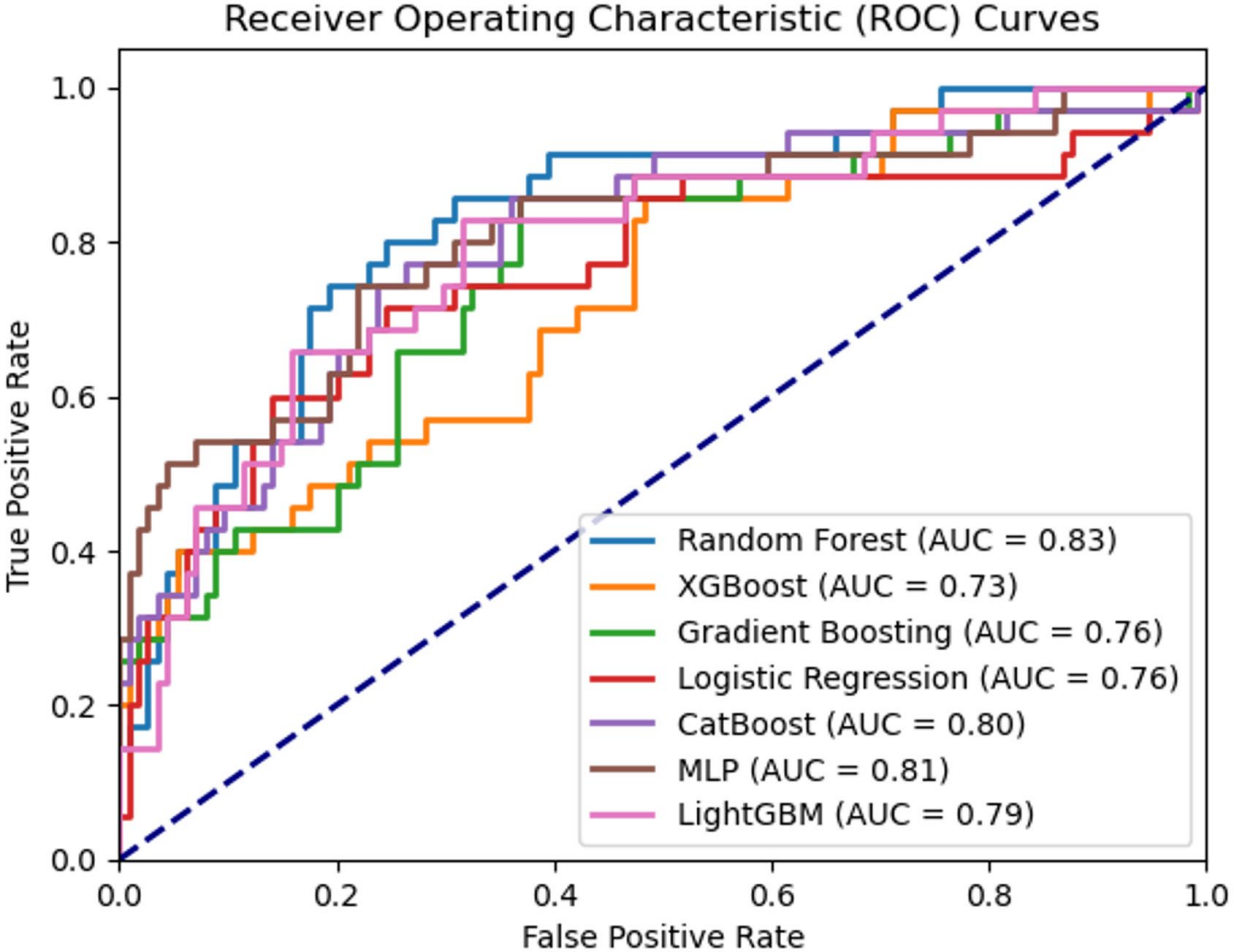
Comparison of model performance, displaying the AUROC curves across the full range of specificity and sensitivity thresholds.

The predictive performance of the machine learning models was comprehensively evaluated through ROC curve analysis. The AUC serves as a robust metric to assess the models’ ability to discriminate between classes, with higher values indicating superior classification performance. As illustrated in Fig. 1, the random forest algorithm exhibited the highest AUC of 0.83, demonstrating remarkable efficacy in achieving an optimal balance between sensitivity and specificity for the classification task. Closely following were the MLP and CatBoost models, with AUC values of 0.81 and 0.80, respectively. These models exhibited strong generalization capabilities across a wide range of threshold settings, indicating their robustness and potential for practical applications. While the XGBoost and gradient boosting algorithms presented relatively lower AUC values of 0.73 and 0.76, respectively, potentially attributable to overfitting or suboptimal parameter tuning, their performance was still respectable. The logistic regression model, with an AUC of 0.76, provided a reasonable baseline for the classification task, albeit it was outperformed by the ensemble methods.

Overall, the ensemble learning techniques, particularly random forest, demonstrated superior predictive performance in this scenario. This can be attributed to their ability to leverage multiple base learners, effectively mitigating individual model biases and enhancing overall accuracy and generalization capabilities. The CatBoost model demonstrated the overall best performance in predicting postoperative outcomes in colon cancer patients, achieving the highest accuracy and AUROC among the evaluated models. To offer more granular insight into classifier performance, particularly for the most effective model identified in our study, the confusion matrix for the CatBoost algorithm is presented in Table 4. Moreover, The CNN achieved the lowest performance with an AUC of 0.70, indicating that convolutional models are less suitable for small, structured clinical datasets compared to ensemble methods.

**Table 4:** Confusion matrix for the catboost model.

Predicted\actual	Dead (positive)	Alive (negative)
Dead	90	49
Alive	83	542

### Feature importance analysis using SHAP values

**Fig. 2:**
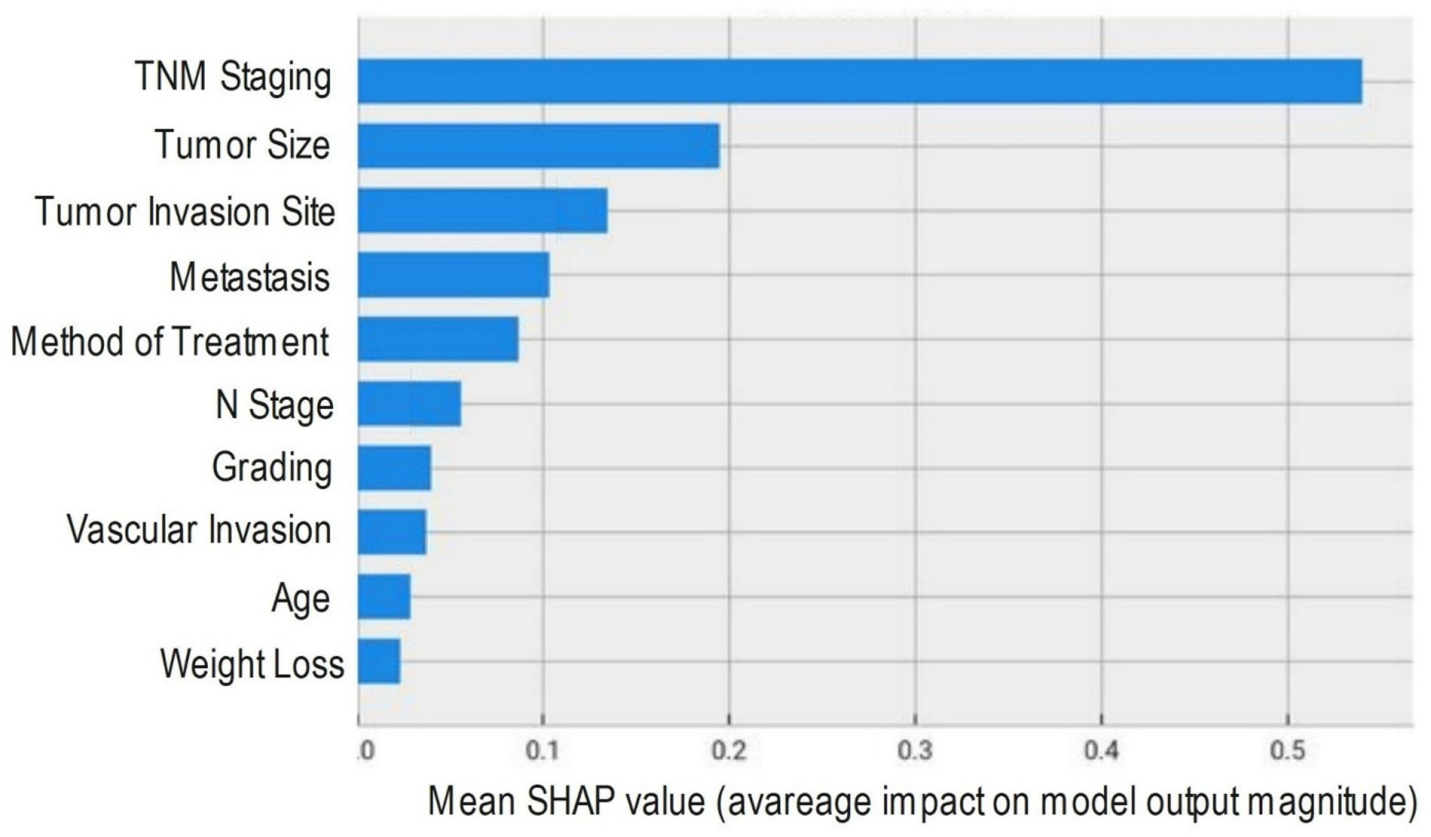
Top ten most influential features for predicting colon cancer.

To provide comprehensive insight into the factors driving our predictive models and to enhance clinical interpretability, we performed Shapley Additive exPlanations (SHAP) value analysis on the top-performing CatBoost model. SHAP values offer a unified and consistent measure of feature importance by calculating the contribution of each feature to the prediction for every instance on the basis of principles from cooperative game theory. On the basis of this analysis, the top ten most influential features for predicting colon cancer treatment outcome were identified and ranked (Fig. 2).

## Discussion

In this study, machine learning models were developed using colon cancer data from the Colorectal Cancer Research Center at Shiraz University of Medical Sciences to identify important prognostic factors for postoperative outcomes. The analysis focused on multiple algorithms, including random forest, XGBoost, gradient boosting, logistic regression, CatBoost, MLP, 1D-CNN, and LightGBM. Among these, CatBoost demonstrated the highest overall accuracy and AUROC, indicating its strong potential for clinical application in predicting patient outcomes. The findings of this study offer valuable insights into the application of machine learning algorithms for predicting survival outcomes in colon cancer patients. The varying performance of the ML models underscores the importance of selecting appropriate modeling techniques and features to optimize predictive accuracy and clinical utility.

CatBoost achieved the highest accuracy and AUROC, highlighting its efficacy in handling complex datasets with mixed data types. This model’s superior performance can be attributed to its ability to effectively process categorical features and mitigate overfitting through robust parameter tuning. The random forest, with the highest AUROC, also proved to be a reliable model, demonstrating a balanced sensitivity and specificity, which is crucial for medical decision-making. The predictive power of logistic regression, particularly in terms of recall, underscores its ability to identify true positive cases effectively. This is essential in clinical scenarios where the cost of missing a positive case can be high. XGBoost and gradient boosting, although slightly less effective, still presented respectable AUROC values, suggesting their potential utility with further optimization. However, the imbalance between precision and recall observed in some classifiers is largely attributable to the skewed distribution of alive versus dead outcomes in our dataset. Despite hyperparameter tuning and balancing measures, traditional models such as random forest and MLP still achieve relatively low recall. This emphasizes the challenge of minority class detection in survival prediction tasks.

Recently, reported evidence has revealed that the increased survival rates among colon cancer patients are due primarily to advancements in treatment^19,20^. This highlights that effective treatment plays a vital role in determining the survival chances of individuals diagnosed with colon cancer. The location, distance and number of metastasis sites are also independent prognostic factors for colon cancer patient survival^21^. Therefore, diverse treatment strategies for patients with different metastatic patterns are needed. Consistent with prior studies, features such as tumor TNM stage, grade, tumor size, method of diagnosis, and surgical approach were shown to be crucial predictors that influence survival and the performance of ML algorithms^22^. In this context, surgery, as a treatment option, has become a significant factor, potentially indicating smaller, operable tumors. In contrast, primary chemotherapy is primarily used for locally advanced colon cancer patients. The method of diagnosis and type of surgery are crucial prognostic factors for colon cancer treatment outcomes. According to Grass et al.^23^, early and accurate surgery of tumors was positively associated with a higher survival rate in nonmetastatic colon cancer patients. Therefore, the diagnostic method plays a vital role in estimating the survival rate of breast cancer patients. The total lymph node (TLN) count also appeared to be an important prognostic factor, which is consistent with other studies that identified the TLN as a significant indicator of colon cancer prognosis^24–26^. Other variables, including tumor TNM stage, grade and tumor size, are crucial predictors of colorectal cancer survival and mortality rates and have potential effects on the performance of ML algorithms, such as accuracy and precision parameters^27^. A previous study used TNM staging (tumor, node, and metastasis) to investigate the prediction of tumor stage in colon cancer patients and reported that the tumor size variable might be a potential prognostic factor, and the random forest model outperformed all other algorithms, with an accuracy of 84% and an AUC of 82% for predicting five-year disease-free survival^11^.

Our results also revealed that performing the ML algorithm, CatBoost, on tumor-related variables alongside patient-related variables had the highest overall accuracy and AUROC, which indicates its potential for clinical application in predicting patient outcomes. The evaluation of model performance through multiple metrics, accuracy, precision, recall, specificity, F1 score, and AUROC, provided a robust framework for assessing the strengths and weaknesses of each algorithm. This multidimensional evaluation is crucial in medical research, where the implications of predictions extend beyond mere statistical accuracy. This multidimensional evaluation of algorithms revealed that the random forest algorithm and XGBoost might be potential algorithms for predicting colon cancer patient outcomes. The inclusion of features such as cancer stage, tumor size, tumor invasion site, treatment type, and clinical symptoms aligns with previous research findings^28–30^. In this context, SHAP value analysis of the top-performing CatBoost model revealed that TNM stage was the feature with the greatest impact on the output performance of the ML model. However, the combination of cancer-related variables with patient demographic variables is pivotal for enhancing the prediction performance of the ML model. Our results revealed that the next most influential variables, in descending order of importance, were tumor size, tumor invasion site, presence of metastasis, method of treatment, N stage, tumor grade, vascular invasion, patient age, and weight loss. This ranking demonstrates that combining tumor-specific characteristics with key demographic and clinical factors substantially enhances the predictive power of machine learning models for survival outcomes in patients with colon cancer.

In line with our findings, several recent studies have suggested that combining diverse data types can significantly improve the prediction accuracy for colorectal cancer outcomes. In this context, combining clinical parameters with gene expression data can increase the accuracy of the ML model^14,15^. Furthermore, a variety of machine learning classifiers, including random forest, XGBoost, and CatBoost, have achieved accuracies between 80% and 93% in similar contexts^15,16^. The use of comprehensive datasets, including data from the Cancer Genome Atlas or other large public cohorts, has been shown to support more robust training and validation of ML-based models to increase model performance in predicting cancer patient outcomes^31^. These findings have meaningful implications for medical practice. By confirming that models such as CatBoost and random forest can reliably identify high-risk patients via accessible clinical data, this study supports the use of ML-based decision-support systems in routine oncology workflows. In practice, these models could assist clinicians in stratifying patients by risk, planning closer monitoring for high-risk patients, and personalizing treatment plans on the basis of predicted survival outcomes. Additionally, the identified key prognostic factors align with clinical guidelines, making the model outputs interpretable and trustworthy for practitioners. Therefore, integrating these models with electronic health record systems could enable real-time risk prediction and continuous refinement as more data become available, ultimately helping to deliver more personalized, timely, and effective care for colon cancer patients.

## Conclusion

In this study, we developed and compared seven machine learning algorithms using postoperative colon cancer data to identify key prognostic factors and predict survival outcomes. CatBoost and random forest achieved the highest accuracy and AUROC, demonstrating robustness in handling categorical variables and mitigating overfitting. Evaluating models across multiple metrics ensured balanced performance assessment, whereas SHAP-based feature importance highlighted clinically meaningful predictors such as TNM staging, tumor size, invasion site, and metastasis. However, the reliance on a single-center retrospective dataset and residual class imbalance may limit generalizability, and the exclusion of genomic or imaging data constrains predictive power. Future work should validate these models in multicenter settings, expand feature sets, and integrate cost-sensitive learning and advanced interpretability methods to increase their clinical impact.

### Limitations

Despite its strengths, this study has several limitations. First, the retrospective design may introduce biases inherent to historical data collection practices, such as selection and information bias, which could affect the reliability of the input data and model outputs. Second, the dataset was sourced from a single research center, potentially limiting the generalizability of the findings to broader or more diverse patient populations. Third, while class imbalance was addressed via techniques such as stratified sampling and SMOTE, some models still exhibited low recall for the minority class, indicating the need for further improvements in sensitivity for rare outcomes. Fourth, although feature importance was analyzed and visualized, the lack of advanced interpretability tools such as SHAP explanations for individual predictions could limit immediate clinical adoption. Finally, the study did not integrate additional data types, such as genomic, imaging, or multiomics information, which could further increase the prediction accuracy and provide deeper insights into patient outcomes. Future research should aim to validate these models in larger, multicenter datasets, incorporate richer data sources, and adopt more advanced model interpretation methods to make AI-driven predictions more transparent and clinically actionable.

## Supplementary Information

Below is the link to the electronic supplementary material.

Supplementary Material 1

## Data Availability

Data are available from the corresponding author upon reasonable request.

## References

[1] Xi, Y. & Xu, P. Global colorectal cancer burden in 2020 and projections to 2040. *Translational Oncol.* **14** (10), 101174 (2021). 10.1016/j.tranon.2021.101174 PMC8273208 34243011

[2] Arnold, M. et al. Global patterns and trends in colorectal cancer incidence and mortality. *Gut* **66** (4), 683–691 (2017). 26818619 10.1136/gutjnl-2015-310912

[3] Gandomani, H. S. et al. Colorectal cancer in the world: Incidence, mortality and risk factors. *Biomedical Res. Therapy*. **4** (10), 1656–1675 (2017).

[4] Iqbal, M. J. et al. Clinical applications of artificial intelligence and machine learning in cancer diagnosis: Looking into the future. *Cancer Cell Int.* **21** (1), 270 (2021). 34020642 10.1186/s12935-021-01981-1 PMC8139146

[5] Cruz, J. A. & Wishart, D. S. Applications of machine learning in cancer prediction and prognosis. *Cancer Inform.* **2**, 117693510600200030 (2006). PMC2675494 19458758

[6] Eisenstein, S., Stringfield, S. & Holubar, S. D. Introduction to big data in colorectal surgery: Using the National Surgical Quality Improvement Project (NSQIP) to perform clinical research in colon and rectal surgery. *Clin. Colon Rectal Surg.* **32** (1), 41 (2019). 30647545 10.1055/s-0038-1673353 PMC6327746

[7] Petrus, A. *An Application of Survival Analysis on the Prevalence and Risk Factors of Breast Cancer in Namibia* (University of Namibia, 2019).

[8] Pearce, C. B., Gunn, S. R., Ahmed, A. & Johnson, C. D. Machine learning can improve prediction of severity in acute pancreatitis using admission values of APACHE II score and C-reactive protein. *Pancreatology* **6** (1–2), 123–131 (2006). 16327290 10.1159/000090032

[9] Das, S., Nayak, S. P., Sahoo, B. & Nayak, S. C. Machine learning in healthcare analytics: A state-of-the-art review. *Arch. Comput. Methods Eng.* 1–40 (2024).

[10] Navin, K., Nehemiah, H. K., Nancy Jane, Y. & Veena Saroji, H. A classification framework using filter–wrapper based feature selection approach for the diagnosis of congenital heart failure. *J. Intell. Fuzzy Syst.* **44** (4), 6183–6218 (2023).

[11] Gupta, P. et al. Prediction of colon cancer stages and survival period with machine learning approach. *Cancers* **11** (12), 2007 (2019). 31842486 10.3390/cancers11122007 PMC6966646

[12] Mamdouh, A., El-Melegy, M. T., Ali, S. A. & El-Baz, A. S. (eds) Prediction of The Gleason Group of Prostate Cancer from Clinical Biomarkers: Machine and Deep Learning from Tabular Data. 2022 International Joint Conference on Neural Networks (IJCNN); : IEEE. (2022).

[13] Li, Y. et al. Optimizing a interpretable diagnostic model for colorectal cancer based on Yin deficiency pattern characteristic genes using 21 machine learning algorithms and bayesian opitimization. (2023).

[14] Mısırlıoğlu, H. K., Leblebici, A., Koçal, G. Ç., Ellidokuz, H. & Başbınar, Y. AI-assisted survival prediction in colorectal cancer: A clinical decision support tool. *J. Basic. Clin. Health Sci.* **8** (3), 771–778 (2021).

[15] Woźniacki, A., Książek, W. & Mrowczyk, P. A novel approach for predicting the survival of colorectal cancer patients using machine learning techniques and advanced parameter optimization methods. *Cancers* **16** (18), 3205 (2024). 39335174 10.3390/cancers16183205 PMC11430446

[16] Britto, C. F. (Ed) Prediction of colon cancer disease with the handling of outliers and overfitting through neural network clustering and optimal tuning. In *2023 International Conference on Advances in Computation, Communication and Information Technology (ICAICCIT)*. 23–24 Nov 2023 (2023).

[17] Katz, M. H. Multivariable analysis: A primer for readers of medical research. *Ann. Intern. Med.* **138** (8), 644–650 (2003). 12693887 10.7326/0003-4819-138-8-200304150-00012

[18] Huynh-Thu, V. A., Saeys, Y., Wehenkel, L. & Geurts, P. Statistical interpretation of machine learning-based feature importance scores for biomarker discovery. *Bioinformatics* **28** (13), 1766–1774 (2012). 22539669 10.1093/bioinformatics/bts238

[19] Jullumstrø, E., Wibe, A., Lydersen, S. & Edna, T. H. Colon cancer incidence, presentation, treatment and outcomes over 25 years. *Colorectal Dis.* **13** (5), 512–518 (2011). 20128833 10.1111/j.1463-1318.2010.02191.x

[20] Van Steenbergen, L. et al. Improved survival of colon cancer due to improved treatment and detection: a nationwide population-based study in the Netherlands 1989–2006. *Ann. Oncol.* **21** (11), 2206–2212 (2010). 20439339 10.1093/annonc/mdq227

[21] Wang, J. et al. Metastatic patterns and survival outcomes in patients with stage IV colon cancer: A population-based analysis. *Cancer Med.* **9** (1), 361–373 (2020). 31693304 10.1002/cam4.2673 PMC6943094

[22] Buk Cardoso, L. et al. Machine learning for predicting survival of colorectal cancer patients. *Sci. Rep.* **13** (1), 8874 (2023). 37264045 10.1038/s41598-023-35649-9 PMC10235087

[23] Grass, F. et al. Impact of delay to surgery on survival in stage I-III colon cancer. *Eur. J. Surg. Oncol.* **46** (3), 455–461 (2020). 31806516 10.1016/j.ejso.2019.11.513

[24] Jiang, C. et al. Metastatic lymph node ratio as a prognostic indicator in patients with stage IV colon cancer undergoing resection. *J. Cancer*. **10** (11), 2534 (2019). 31258759 10.7150/jca.29216 PMC6584347

[25] Märkl, B. et al. The clinical significance of lymph node size in colon cancer. *Mod. Pathol.* **25** (10), 1413–1422 (2012). 22684222 10.1038/modpathol.2012.92

[26] Li, Q. et al. Negative to positive lymph node ratio is a superior predictor than traditional lymph node status in stage III colorectal cancer. *Oncotarget* **7** (44), 72290 (2016). 27474167 10.18632/oncotarget.10806 PMC5342162

[27] Dimitriou, N., Arandjelović, O., Harrison, D. J. & Caie, P. D. A principled machine learning framework improves accuracy of stage II colorectal cancer prognosis. *NPJ Digit. Med.* **1** (1), 52 (2018). 31304331 10.1038/s41746-018-0057-x PMC6550189

[28] Jiang, D. et al. A machine learning-based prognostic predictor for stage III colon cancer. *Sci. Rep.* **10** (1), 10333 (2020). 32587295 10.1038/s41598-020-67178-0 PMC7316723

[29] Xu, Y., Ju, L., Tong, J., Zhou, C-M. & Yang, J-J. Machine learning algorithms for predicting the recurrence of stage IV colorectal cancer after tumor resection. *Sci. Rep.* **10** (1), 2519 (2020). 32054897 10.1038/s41598-020-59115-y PMC7220939

[30] Bülbül, H. M., Burakgazi, G. & Kesimal, U. Preoperative assessment of grade, T stage, and lymph node involvement: machine learning-based CT texture analysis in colon cancer. *Japanese J. Radiol.* **42** (3), 300–307 (2024). 10.1007/s11604-023-01502-2 37874525

[31] Nikolaou, N. et al. A machine learning approach for multimodal data fusion for survival prediction in cancer patients. *NPJ Precision Oncol.* **9** (1), 128 (2025). 10.1038/s41698-025-00917-6 PMC12053085 40325104

