# Supplementary material for: Machine learning application in colon cancer treatment outcome prediction
Source: Sci Rep. 2026 Jan 24;16:6159. doi: 10.1038/s41598-026-36917-0 (PMC12905234; doi:10.1038/s41598-026-36917-0)
Supplement: Supplementary file 1 — Supplementary Material 1 [file 41598_2026_36917_MOESM1_ESM.docx]

Supplementary Table 1. Summary of tuned hyperparameters and optimal settings for all models

| **Model** | **Tuned Hyperparameters** | **Search Range** | **Search Strategy** | **Selection Metric** |
| --- | --- | --- | --- | --- |
| **Random Forest** | n_estimators, max_depth, min_samples_split, max_features | 50–500; None, 5–30; 2–10; 'sqrt', 'log2' | Grid + Random Search | 5-fold CV, Max AUROC |
| **XGBoost** | learning_rate, n_estimators, max_depth, subsample, colsample_bytree | 0.01–0.3; 50–500; 3–10; 0.6–1.0; 0.6–1.0 | Random Search | 5-fold CV, Max AUROC |
| **Gradient Boosting** | learning_rate, n_estimators, max_depth, subsample, colsample_bytree | 0.01–0.3; 50–500; 3–10; 0.6–1.0; 0.6–1.0 | Random Search | 5-fold CV, Max AUROC |
| **CatBoost** | learning_rate, depth, l2_leaf_reg, border_count | 0.01–0.3; 4–10; 1–10; 32–255 | Grid + Random Search | 5-fold CV, Max AUROC |
| **LightGBM** | learning_rate, num_leaves, n_estimators, min_child_samples | 0.01–0.3; 20–100; 50–500; 5–50 | Random Search | 5-fold CV, Max AUROC |
| **MLP** | hidden_layer_sizes, activation, alpha, learning_rate_init | (50,), (100,), (100,50); relu/tanh; 1e-4–1e-2 | Grid Search | 5-fold CV, Max AUROC |
| **Logistic Regression** | C, penalty, solver | 0.01–10; l1/l2; liblinear/saga | Grid Search | 5-fold CV, Max AUROC |
| **1D-CNN** | filters, kernel_size, dropout_rate, batch_size, epochs | filters: [32, 64]; kernel_size: [3, 5]; dropout_rate: 0.2–0.5; batch_size: [16, 32, 64]; epochs: 50–150 | Random Search | \| Search \| \| --- \|  \| 5-fold CV, Min Val Loss \| \| --- \| |
